# IRAK-M alters the polarity of macrophages to facilitate the survival of *Mycobacterium tuberculosis*

**DOI:** 10.1186/s12866-017-1095-2

**Published:** 2017-08-23

**Authors:** Pei Shen, Quan Li, Jilei Ma, Maopeng Tian, Fei Hong, Xinjie Zhai, Jianrong Li, Hanju Huang, Chunwei Shi

**Affiliations:** 1 Department of Pathogen Biology, School of Basic Medicine, Tongji Medical College, Huazhong University of Science and Technology, Wuhan, 430030 People’s Republic of China; 2 Department of Clinical Microbiology, School of Public Health, Taishan Medical University, Tai’an, 271016 People’s Republic of China; 3 Wuhan Institute for Tuberculosis Control, Wuhan, 430030 People’s Republic of China

**Keywords:** *Mycobacterium tuberculosis*, IRAK-M, Macrophage, Polarization, Intracellular survival

## Abstract

**Background:**

Intracellular bacterium, *Mycobacterium tuberculosis* (*M. tb*), infects specifically macrophages as host cells. IRAK-M, a member of IRAK family, is a negative regulator in TLR signaling and specifically expresses in monocytes and macrophages. The role of IRAK-M in intracellular growth of *M. tb* and macrophage polarization was explored, for deeply understanding the pathogenesis of *M. tb*, the significance of IRAK-M to innate immunity and pathogen-host interaction.

**Methods:**

IRAK-M expression was detected in *M. tb* infected macrophages and in human lung tissue of pulmonary tuberculosis with immunofluorescence staining, Western blot and immunohistochemistry. IRAK-M knock-down and over-expressing cell strains were constructed and intracellular survival of *M. tb* was investigated by acid-fast staining and colony forming units. Molecular markers of M1-type (pSTAT1 and iNOS) and M2-type (pSTAT6 and Arg-1) macrophages were detected using Western blot in IRAK-M knockdown U937 cells infected with *M. tb* H37Rv. U937 cells were stimulated with immunostimulant CpG7909 into M1 status and then infected with *M. tb* H37Rv. Expression of IRAK-M, IRAK-4 and iNOS was detected with immunofluorescence staining and Western blot, to evaluate the effect of IRAK-M to CpG directed M1-type polarization of macrophages during *M. tb* infection. Molecules related with macrophage’s bactericidal ability such as Hif-1 and phosphorylated ERK1/2 were detected with immunohistochemistry and Western blot.

**Results:**

IRAK-M increased in *M. tb* infected macrophage cells and also in human lung tissue of pulmonary tuberculosis. IRAK-M over-expression resulted in higher bacterial load, while IRAK-M interference resulted in lower bacterial load in *M. tb* infected cells. During *M. tb* infection, IRAK-M knockdown induced M1-type, while inhibited M2-type polarization of macrophage. M1-type polarization of U937 cells induced by CpG7909 was inhibited by *M. tb* infection, which was reversed by IRAK-M knockdown in U937 cells. IRAK-M affected Hif-1 and MAPK signaling cascade during *M. tb* infection.

**Conclusions:**

Conclusively, IRAK-M might alter the polarity of macrophages, to facilitate intracellular survival of *M. tb* and affect Th1-type immunity of the host, which is helpful to understanding the pathogenesis of *M. tb*.

## Background

*Mycobacterium tuberculosis* (*M. tb*) infection is widespread across the globe, which is a severe threat to human health [1]. *M. tb* epidemic is a major issue to be solved in the health field. However, research and development of *M. tb* vaccines are not satisfied. *M. tb* infects specifically macrophages as host cells like *Brucella*, *Salmonella* and other intracellular bacteria [2, 3]. Macrophages are not only the shelters of *M. tb*, but also the key effector cells involved in anti-*M. tb* infection [4].

The functions of activated macrophages depend on the regulation by a variety of signaling pathways, including pattern-recognition receptors (PRRs), leading to the different direction of macrophage polarization [5]. Macrophages primed with Th1 cytokine (IFN-γ) in the presence of microbial ligands, polarize to pro-inflammatory M1-type cells and develop the phenotypes typical of classically activated macrophages (CAM), leading to increased expression of inducible nitric oxide synthase (iNOS) [6, 7]. In contrast, macrophages activated with Th2 cytokines (IL-4, IL-13 or IL-10), polarize to distinct M2 phenotypes, M2a, M2b and M2c, respectively, associated with alternatively activated macrophages (AAM), which display anti-inflammatory, phagocytosis-promoting and tissue-repairing activities [8]. M2 macrophages are characterized by expression of typical markers, including arginase 1 (Arg-1), scavenger and mannose receptors (MR/CD206), anti-inflammatory cytokine IL-10 [7, 9–12]. Activation of M1 type macrophages is promoted by IFN-γ-mediated Janus kinase-signal transducer and activator of transcription 1 (JAK-STAT1) signaling [5]. By contrast, STAT6 is required to drive activation of M2 macrophage during Th2 immune responses in the presence of IL-4 and/or IL-13 [13].

It is noteworthy that intracellular bacteria prefer to utilize macrophages as their gateway and shelter invading into hosts. As key links of PAMP signaling pathway were analyzed, an intriguing molecule, IRAK-M was noticed. IRAK-M, named also as IRAK3, is restricted to express in certain cell types such as monocytes/macrophages and lung epithelial cells [14] and plays a negative role in PAMP-TLR signaling pathway, by means of inhibiting IRAK1/4 phosphorylation and dissociation [15]. IRAK-M belongs to IRAK family, which includes IRAK1/4, IRAK2 and IRAK-M. IRAK1 and IRAK4 are active kinases [16]. Upon stimulation, IRAK1 and IRAK4 phosphorylate and form complexes with TRAF6, to transmit the signaling forward to activate downstream signaling molecules, such as NF-κB, IRF7 and JNK [17]. IRAK-M and IRAK2 have no kinase activity due to the lack of an aspartic acid residue. IRAK-M molecule combines with IRAK1/4 to form the IRAK-M complex, which induces the expression of negative regulators such as SOCS1, SHIP1, A20 and IκBα. In this way, IRAK-M acts as a negative regulator in TLR signaling of monocytes and macrophages to restrict tissue damage upon excessive immune response [18]. It was reported that during *M. tb* infection, IRAK-M was involved in the restriction of Th1 anti-tuberculosis immunity [19–21]. Whether intracellular bacteria such as *M. tb* utilize IRAK-M to direct macrophage polarity and facilitate bacterial intracellular survival, deserves further investigation.

In the current work, IRAK-M expression was detected in *M. tb* infected macrophage cells and also in lung tissue of patients with pulmonary TB. Cell strains of IRAK-M knockdown or over-expression were constructed to study the role of IRAK-M molecule in *M. tb* infection and macrophage polarization. The effect of IRAK-M to some other macrophage regulatory molecules such as Hif-1 and MAPK was studied as well. This work might have important implications for deeply understanding the pathogenesis of *M. tb*, the significance of IRAK-M to innate immunity and pathogen-host interaction as well.

## Methods

### Bacterial strains and cultures

Virulent *Mycobacterium tuberculosis* H37Rv strains (kindly provided by Prof. Xionglin Fan, Huazhong University of Science and Technology, China) were grown in Middlebrook 7H11 agar plates (Difco Laboratories, Sparks, MD, USA), supplemented with 10% ADC and 0.5% glycerol, including ampicillin (100 μg/ml), carbenicillin (50 μg/ml), polymyxin B Sulfate (25 μg/ml) and amphotericin B (2.5 μg/ml), which inhibits the growth of other bacteria and fungi.

### Cell culture, construction of lentiviral vector and infection of lentivirus

Human monocytic leukemia line U937 (ATCC® CRL-1593.2) and human T-cell acute lymphoblastic leukemia line Jurkat (ATCC® TIB-152™) were cultured at 37 °C in CO_2_ incubator (CCL-170B-8, ESCO, Singapore) in RPMI-1640 medium, supplemented with 10% fetal bovine serum, 100 U/ml penicillin and 100 μg/ml streptomycin. U937 cells were stimulated with 20 ng/ml phorbol myristate acetate (PMA) for 24 h and the cells were then allowed to recuperate for 40 h [22–24]. Lentiviral vectors that interferes or over-expresses the expression of human *irak-m*, were constructed via technical support from Shanghai GeneChem (Shanghai, China) and named as LV-IRAK-M-RNAi (target sequence: CCTTGGCACATTCGAATCGGTATAT) or LV-IRAK-M (the full length human *irak-m* cDNA clone, GENE_ID 11213, Genbank No. NM_007199), following the NYMC Institutional Biosafety Committee approval. Jurkat and U937 cells were respectively infected with negative control lentivirus vectors (NC), LV-IRAK-M-RNAi or LV-IRAK-M. U937 cells were plated at a density of 6 × 10^5^ per well in enhanced infection solution (Eni.S) and transfected with lentivirus at MOI (multiplicity of infection) 40. Jurkat cells were plated at a density of 6 × 10^5^ per well in enhanced infection solution (Eni.S) with 5 μg/ml polybrene and transfected with lentivirus at MOI 50. Eni.S medium was replaced with complete RPMI-1640 medium after 12 h. Microscopy of green fluorescence was performed to monitor the expression of lentivirus. Infection efficiency of viral vector and positive cell proportion were determined by manual cell count.

### CpG7909 stimulation and H37Rv challenge of cells

The cells were cultured at 37 °C in CO_2_ incubator in RPMI-1640 medium, supplemented with 10% fetal bovine serum, free of penicillin and streptomycin. Cells were plated at a density of 6 × 10^5^ per well and stimulated with CpG7909 at concentration of 0.5 or 2 μg/ml for 24 h. Virulent *M. tuberculosis* H37Rv strain was used to infect cells at MOI 10. To inactivate virulent bacteria, 1 mg colonies of H37Rv were resuspended in 100 μl PBS and boiled at 100 °C for 10 min. Five hours after challenge, cells were washed three times with PBS and resuspended in complete RPMI-1640 medium [25, 26].

### Acid-fast staining and colony counting

1 × 10^6^ cells were collected at 5 or 24 h post challenge and the supernatant was rejected. Cells were washed three times with PBS and fixed in 4% para-formaldehyde for 15 min on the slides. Acid-fast (AF) staining was performed as previously described [27] and the slides were observed under a light microscope with no prior knowledge of grouping and cell treatment. For colony counting, 1 × 10^5^ cells were washed aseptically, homogenized and plated at 10-fold serial dilutions on Middlebrook 7H11 agar for determination of CFU of virulent *M. tuberculosis* H37Rv. Plates were incubated at 37 °C for 3 to 4 weeks and visible colonies were counted to evaluate bacterial load, which was represented as Log10 CFU ± SEM for each group (*n* = 3).

### Immunofluorescent staining and microscopy

Cells were fixed in 4% para-formaldehyde for 15 min, permeabilized with 0.1% Triton X-100 in PBS for 10 min, and counter-stained with antibodies against IRAK-M (4369, Cell signaling, USA) or IRAK4 (4363, Cell signaling, USA). The coverslips were mounted onto microscope slides in Anti-fade Mounting Medium (Beyotime, China). Fluorescent images were visualized and captured using Olympus BX51 upright fluorescent microscope (Olympus, Japan).

### Immunohistochemistry

Six pairs of peripheral tissues from lung squamous cell carcinoma or lung adenocarcinoma, which were regarded as negative control, and tissues from human pulmonary tuberculosis were collected from surgical excision (Wuhan Institute for Tuberculosis Control, China). The formalin-fixed and paraffin-embedded tissues were respectively cut into 4-μm sections and then deparaffinized routinely. The slides were incubated with antibodies (Hif-1α, 2015–1, Epitomics, USA; IRAK-M, 4369, Cell signaling, USA), then washed with PBS and incubated with Envision™ (DAKO, Shanghai, China; polyperoxidase-anti-mouse/rabbit IgG). After washing, the slides were colored with 3,3-diaminobenzidine and counter-stained with haematoxylin.

### Western blot

Cells were washed twice with PBS and resuspended in lysis buffer (50 mM Tris–HCl pH 8.0, 1 mM EDTA, 250 mM NaCl, 1% NP-40 and 0.5% Na-Deoxycholate). Cell lysates were then shaken for 30 min on an orbital shaker at 4 °C and centrifuged for 20 min at 12,000×g and the protein containing supernatant was collected. Protein concentration of cell lysates was estimated using a commercial kit (Bio-Rad, USA). SDS-PAGE and Western blot were performed to determine the expression of Arg-1 (GTX109242, Gene Tex, USA), iNOS (GTX31048, Gene Tex, USA), Hif-1α (2015–1, Epitomics, USA), IRAK1 (4504, Cell signaling, USA), IRAK2 (4367, Cell signaling, USA), IRAK4 (4363, Cell signaling, USA), IRAK-M (4369, Cell signaling, USA), pERK1/2 (2219–1, Epitomics, USA), pSTAT1 (7649, Cell signaling, USA), pSTAT6 (9361, Cell signaling, USA), STAT1 (10144–2-AP, Proteintech, USA), STAT6 (51073–1-AP, Proteintech, USA), VEGF (sc-7269, Santa cruz, USA), β-actin (M2010S, Abmart, China).

### Statistical analysis

All statistical analysis was performed using SPSS Graduate Pack 11.0 statistical software (SPSS). One-way ANOVA analysis or Student’s t test was used to compare the mean of each treatment, with a *P*-value of less than 0.05 considered statistically significant.

## Results

### IRAK-M increased in *M. tb* infected macrophage cells and also in human lung tissue of pulmonary tuberculosis

**Fig. 1:**
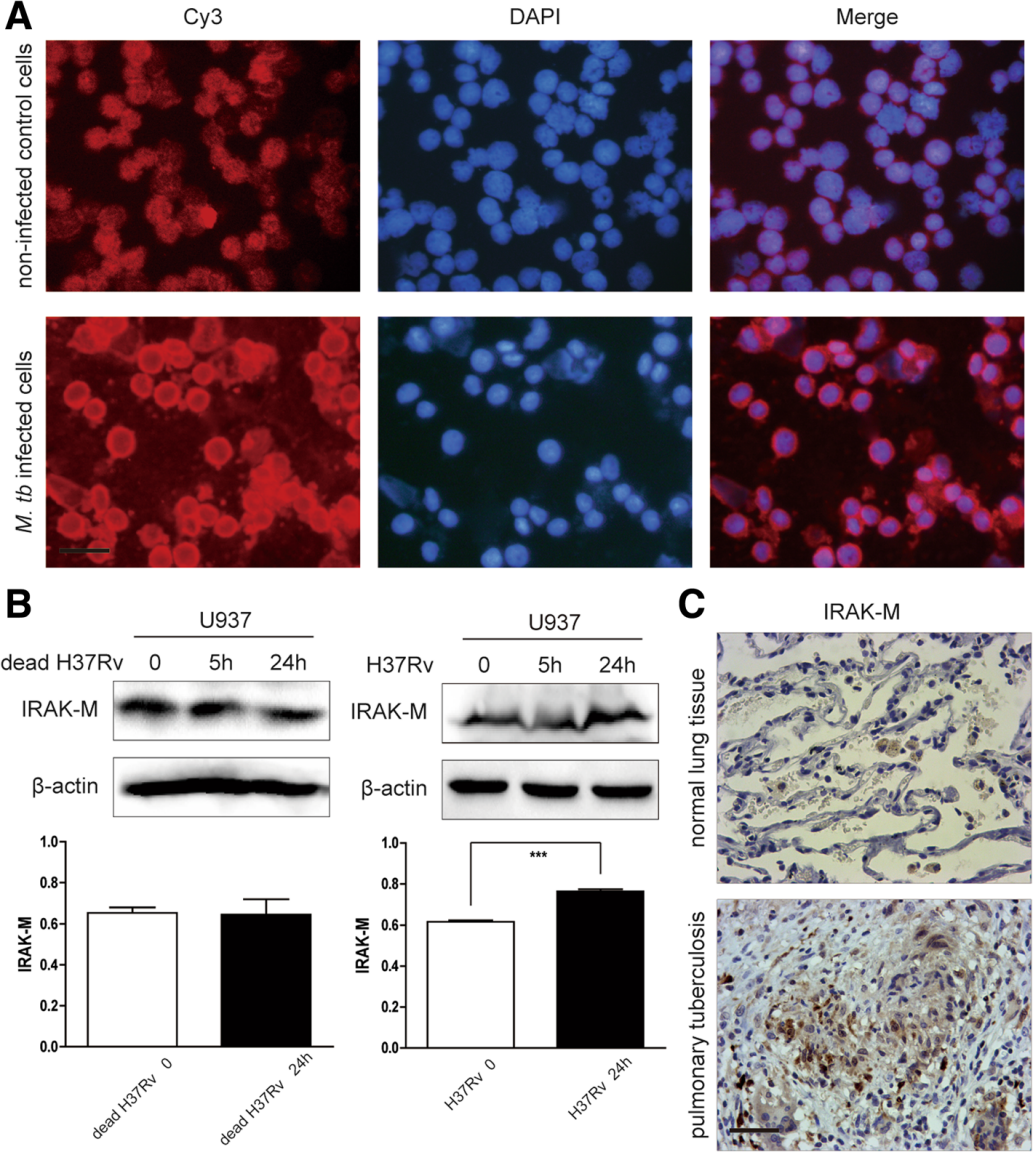
IRAK-M increased in *M. tb* infected macrophage cells and also in *M. tb* infected lung tissue. *M. tb* induced increased expression of IRAK-M in U937 cells. U937 cells were infected with *M. tb* H37Rv (MOI 10) for 24 h after seeding. **a** Cells grown on the coverslips were stained with anti-IRAK-M by immunocytochemistry. Representative images from three experiments were shown as merged results of IRAK-M (Cy3, red) and DAPI (blue) (1000×, scale bar 10 μm). **b** Expression of IRAK-M in U937 cells infected by inactivated or living *M. tb* H37Rv (MOI 10) was evaluated by Western blot. Densitometric analysis of IRAK-M expression was performed using pooled data from three such experiments. Results were expressed as Mean ± SD, ***: *P* < 0.001. **c** IRAK-M expression was increased in tissue of pulmonary tuberculosis. Human samples from pulmonary tuberculosis (*n* = 6) were collected for IRAK-M detection by immunohistochemistry (400×, scale bar 50 μm). Para-carcinoma tissues of human lung cancer (*n* = 6), were used as negative control. Representative images were shown

Macrophage has been demonstrated to be central effector cell of innate immune response against *M. tb* [9, 13] and we were particularly interested in IRAK-M molecule, which is a negative regulator of TLR signaling and specifically expresses in monocytes and macrophages. Expression levels of IRAK-M in U937 cells infected with virulent *M. tb* strain H37Rv were determined by immunocytochemistry and Western blot. As expected, U937 cells exhibited increased IRAK-M expression at 24 h of H37Rv infection, as compared with non-infected control cells (Fig. 1a and b). Interestingly, no increase of IRAK-M was found in U937 cells infected with dead *M.tb* H37Rv (Fig. 1b), which indicated that only the active *M. tb* induces the expression of IRAK-M.

Histological expression of IRAK-M in *M. tb* infection was further determined in samples from human pulmonary tuberculosis, compared with para-carcinoma tissues of lung cancer as negative control. It was shown that IRAK-M expression was increased in pulmonary tuberculosis in comparison with normal lung tissue (Fig. 1c). Collectively, these results indicated that *M. tb* infection induced increased expression of IRAK-M.

### IRAK-M over-expression induced higher bacterial load, while IRAK-M knockdown resulted in lower bacterial load in *M. tb* infected cells

**Fig. 2:**
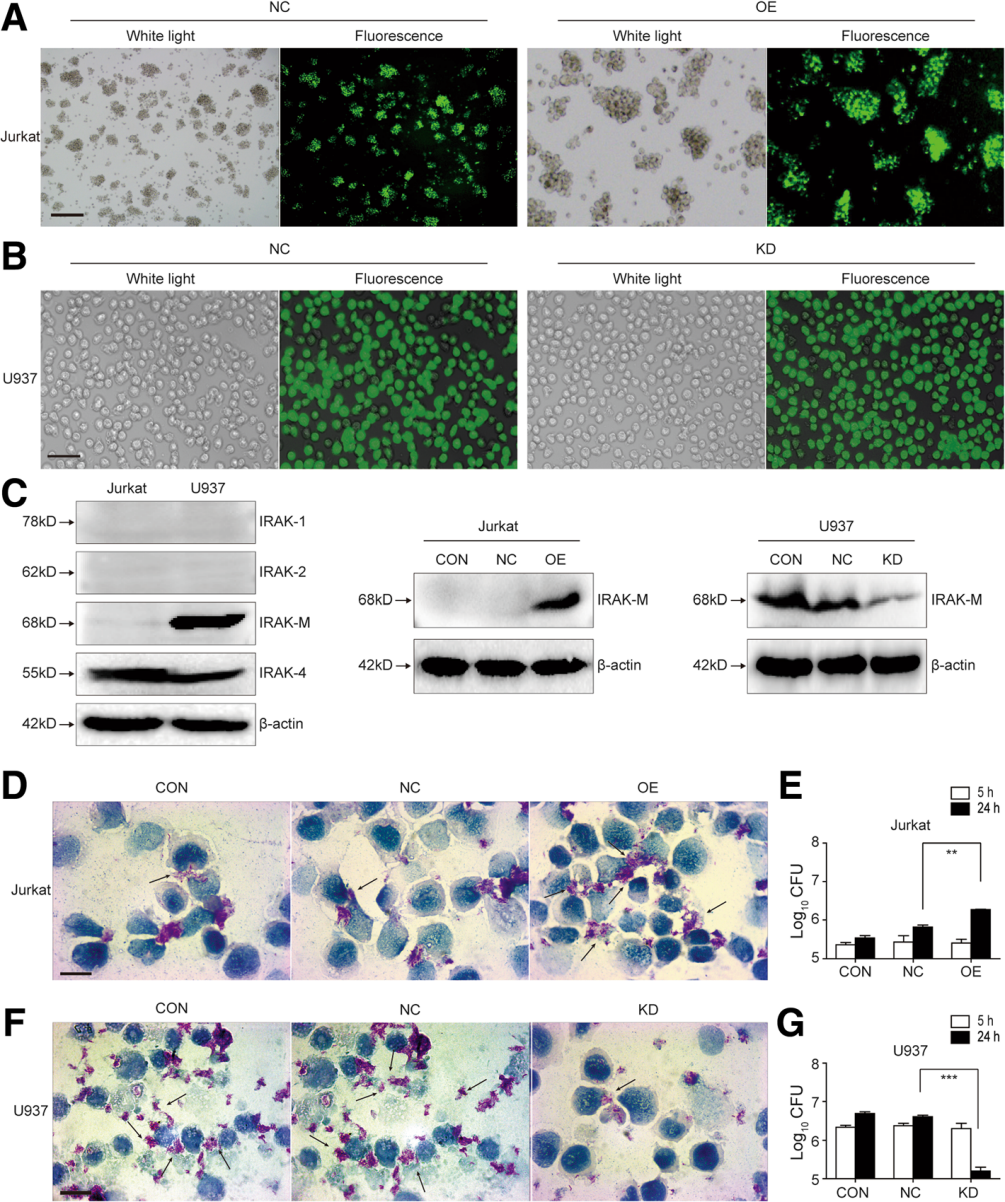
IRAK-M over-expression resulted in higher bacterial load, while IRAK-M interference resulted in lower bacterial load in *M. tb* infected cells. Lentiviruses that over-expresses (IRAK-M-GFP-Lentivirus, OE) or interferes with (IRAK-M-RNAi-GFP-Lentivirus, KD) IRAK-M expression, were constructed. **a** Lentiviruses of negative control (NC) and OE were introduced into Jurkat cells. Cells were examined by light microscope and fluorescent microscope at 96 h post-transfection. More than 80% of Jurkat cells expressed GFP (200×, scale bar 50 μm). **b** Lentiviruses of NC and KD were introduced in U937 cells. Cells were examined by light microscope and fluorescent microscope at 96 h post-transfection. More than 90% of U937 cells expressed GFP (200×, scale bar 100 μm). **c** Expression of IRAK1-4 in Jurkat and U937 cells was evaluated by Western blot. **d**-**g** Bacterial load in Jurkat (**d**, **e**) and U937 cells (**f**, **g**) challenged with virulent *M. tuberculosis* H37Rv strain (MOI 10) were analyzed by acid-fast staining and colony forming units (CFU). Cells were infected with lentivirus for 96 h, challenged with H37Rv for 5 h and washed three times with PBS. **d**, **f** At 24 h post-infection, 1 × 10^6^ cells were resuspended in 20 μl PBS and fixed in 4% para-formaldehyde for 15 min on the slides. Acid-fast staining (AF) was performed and *arrows* indicated AF-positive bacteria in the cells (1000×, scale bar 10 μm). **e**, **g** For determination of CFU, at 5 and 24 h post-infection, 1 × 10^5^ cells were washed aseptically, homogenized and plated at 10-fold serial dilutions on Middlebrook 7H11 agar. CFU numbers per plate were counted to evaluate the bacterial load 3 to 4 weeks later. The bacterial load in cells of different groups was expressed as Log10 CFU ± SEM (**, *p* < 0.01; ***, *p* < 0.001)

To investigate the effects of IRAK-M to *M. tb* intracellular survival, IRAK-M over-expression was constructed in Jurkat cells (OE), a lymphoblastic leukemia cell line, which does not express IRAK-M (Fig. 2c). IRAK-M knockdown system was constructed in U937 cells (KD), which has abundant expression of IRAK-M (Fig. 2c). Both cell lines have endogenous expression of IRAK4, but no expression of IRAK1 and IRAK2 (Fig. 2c). Jurkat and U937 cells were successfully infected by lentivirus, to accomplish in vitro over-expression or knockdown of IRAK-M molecule (Fig. 2a–c).

To further investigate whether IRAK-M affected intracellular survival of *M. tb*, virulent *M. tb* H37Rv strain was used to challenge cells. Acid-fast staining and colony forming units were performed to determine living bacteria in Jurkat and U937 cells with different IRAK-M expression level at 5 or 24 h post infection. There was no variation of CFU count within groups in both Jurkat cells and U937 cells at 5 h post infection (Fig. 2e and g), indicating no variation of entry start of bacteria into cells. However, at 24 h post infection, in Jurkat cells, over-expression of IRAK-M led to increase of bacterial loads, both in acid-fast staining (Fig. 2d) and CFU count (Fig. 2e), as compared with control groups CON and NC (Fig. 2d and e). While in U937 cells, knockdown of IRAK-M led to inhibition of bacterial proliferation (Fig. 2f and g). Intracellular survival of *M. tb* was also analyzed in IRAK-M over-expressed U937 cells. However, as U937 cells have abundant endogenous expression of IRAK-M, cells with exogenously-introduced IRAK-M via lentivirus did not show any significant difference with parental cells (data not shown). The above results in IRAK-M knockdown and over-expression cell systems implied that IRAK-M expression is essential to intracellular survival and proliferation of H37Rv.

### IRAK-M knockdown induced M1-type, while inhibited M2-type polarization of macrophage, during *M. tb* infection

**Fig. 3:**
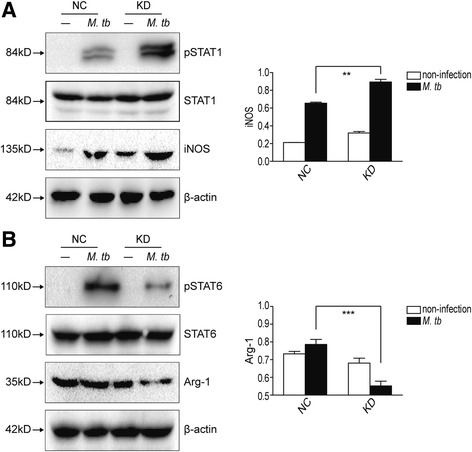
IRAK-M knockdown induced M1-type, while inhibited M2-type polarization of macrophage, during *M. tb* infection. U937 cells were infected with NC or KD lentivirus for 96 h, challenged with H37Rv (MOI 10) for 24 h. Cells were resuspended in lysis buffer and Western blot was performed to detect STAT1, phosphorylated STAT1 (pSTAT1), iNOS (**a**), STAT6, phosphorylated STAT6 (pSTAT6) and Arg-1 (**b**). Densitometric analysis was performed using pooled data from three such experiments. Data were mean ± SD (**, *p* < 0.01; ***, *p* < 0.001)

To study whether intracellular survival of H37Rv affected by IRAK-M is correlated with polarization of macrophages, molecular markers of macrophage polarization, such as phosphorylated STAT1/6 and iNOS/Arg-1 were detected in H37Rv challenged U937 cells. Because of the T cell nature but not macrophage nature of Jurkat cells, polarization markers were not detected in Jurkat cells, while only in U937 cells. It was shown that during H37Rv infection, M1-type markers, phosphorylated STAT1 and iNOS, and M2-type markers, phosphorylated STAT6 and Arg-1, were increased in varying degree (Fig. 3a and b), indicating that *M. tb* infection induced both M1- and M2-type polarization of macrophages. Knockdown of IRAK-M resulted in an increase of phosphorylated STAT1 and iNOS (Fig. 3a) and inhibition of phosphorylated STAT6 and Arg-1 (Fig. 3b), implying that IRAK-M knockdown promoted M1-type, but inhibited M2-type polarization of macrophages.

### M1-type polarization of U937 cells induced by CpG7909 was inhibited by *M. tb* infection, which can be reversed by IRAK-M knockdown in U937 cells

**Fig. 4:**
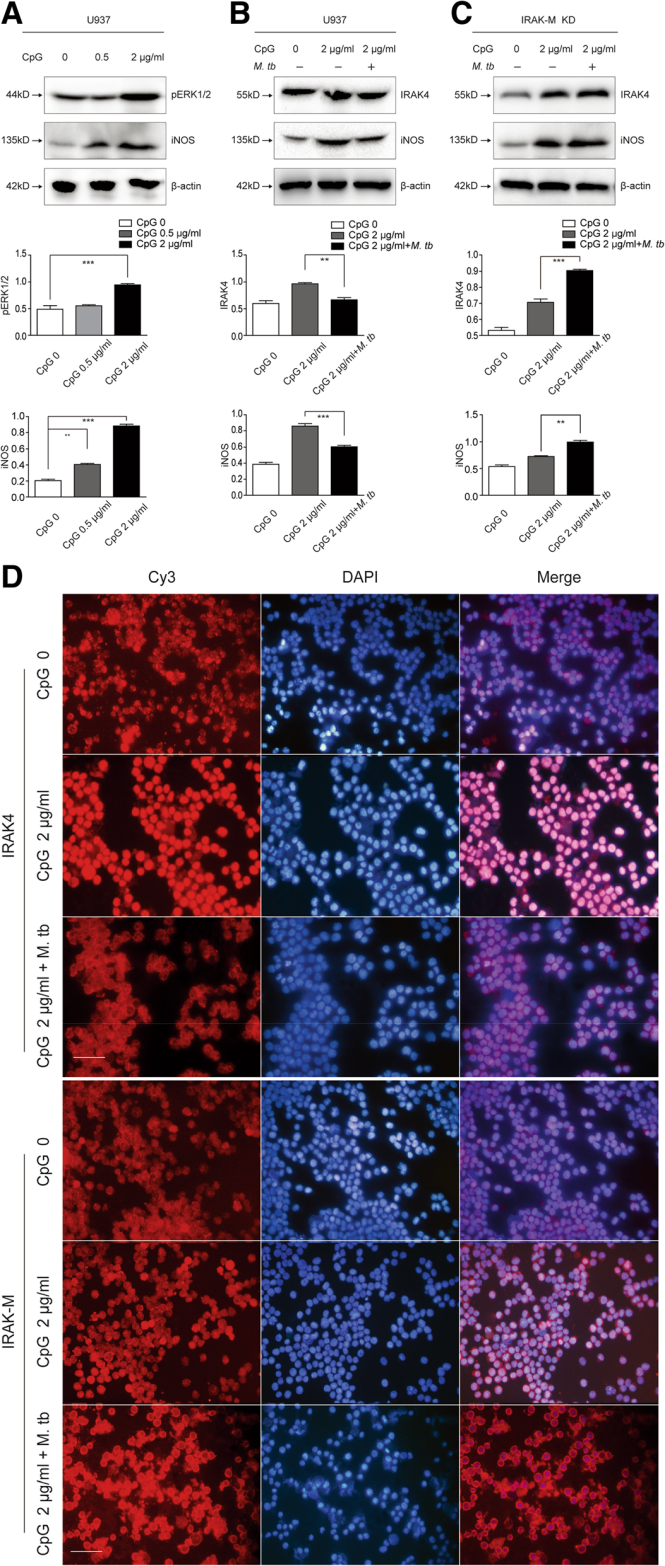
M1-type polarization of U937 cells induced by CpG7909 was inhibited by *M. tb* infection, which can be reversed by IRAK-M knockdown in U937 cells. **a** 2 μg/ml CpG7909 induced M1-type polarization of U937 cells. U937 cells were stimulated with CpG7909 (0, 0.5 μg/ml and 2 μg/ml) for 24 h and 50 μg of cell lysates was analysed by Western blot to detect the expression of phosphorylated ERK1/2 and iNOS. Densitometric analysis was performed using pooled data from three such experiments. Data were mean ± SD (**, *p* < 0.01; ***, *p* < 0.001). **b** *M. tb* infection inhibited M1-type polarization of U937 cells induced by CpG7909. U937 cells were stimulated with CpG7909 (2 μg/ml) for 24 h, then challenged with H37Rv (MOI 10) for 24 h. Western blot was performed to detect IRAK4 and iNOS. Densitometric analysis was performed using pooled data from three such experiments. Data were mean ± SD (**, *p* < 0.01; ***, *p* < 0.001). **c** IRAK-M knockdown rescued M1-type polarization of U937 cells induced by CpG7909, which was inhibited by *M. tb* infection. U937 cells were infected with IRAK-M KD lentivirus for 96 h, stimulated with CpG7909 (2 μg/ml) for 24 h, challenged with H37Rv (MOI 10) for 24 h. Fifty microgram of cell lysates was analysed by Western blot to detect IRAK4 and iNOS. Densitometric analysis was performed using pooled data from three such experiments. Data were mean ± SD (**, *p* < 0.01; ***, *p* < 0.001). **d** IRAK-4 and IRAK-M expression in CpG-stimulated and *M. tb-*infected U937 cells. U937 cells grown on the coverslips were stimulated with CpG7909 (2 μg/ml) for 24 h, then infected with H37Rv (MOI 10) for 24 h, co-stained with anti-IRAK4 (Cy3) or anti-IRAK-M (Cy3) and DAPI (blue) by immunocytochemistry (400×, scale bar 50 μm)

In the current work, the ligand of TLR9, CpG7909 was used as immunostimulant to direct U937 cells firstly into M1 status and U937 cells were then infected with *M. tb* H37Rv strain. The increase of phosphorylated ERK1/2 (pERK1/2) (Fig. 4a), iNOS (Fig. 4a) and IRAK-4 (Fig. 4d) indicated that 2 μg/ml CpG7909 induced activation and M1-type polarization of U937 cells. Virulent *M. tb* H37Rv strain was then used to challenge U937 cells stimulated with 2 μg/ml CpG7909. The increase of IRAK-M (Fig. 4d), reduction of iNOS (Fig. 4b) and IRAK4 (Fig. 4b and d) in infected U937 cells, indicated that *M. tb* infection resulted in an increase of IRAK-M and inhibition of CpG7909-induced M1-type polarization of U937 cells. When IRAK-M was knocked down in U937 cells, the decreased expression of iNOS and IRAK4, due to *M. tb* infection, was reversed in U937 cells upon CpG stimulation (Fig. 4c). Overall, all of these results suggested that IRAK-M might be utilized by *M. tb* to regulate the direction of macrophage’s polarization.

### IRAK-M affected Hif-1 and MAPK signaling cascade during *M. tb* infection

**Fig. 5:**
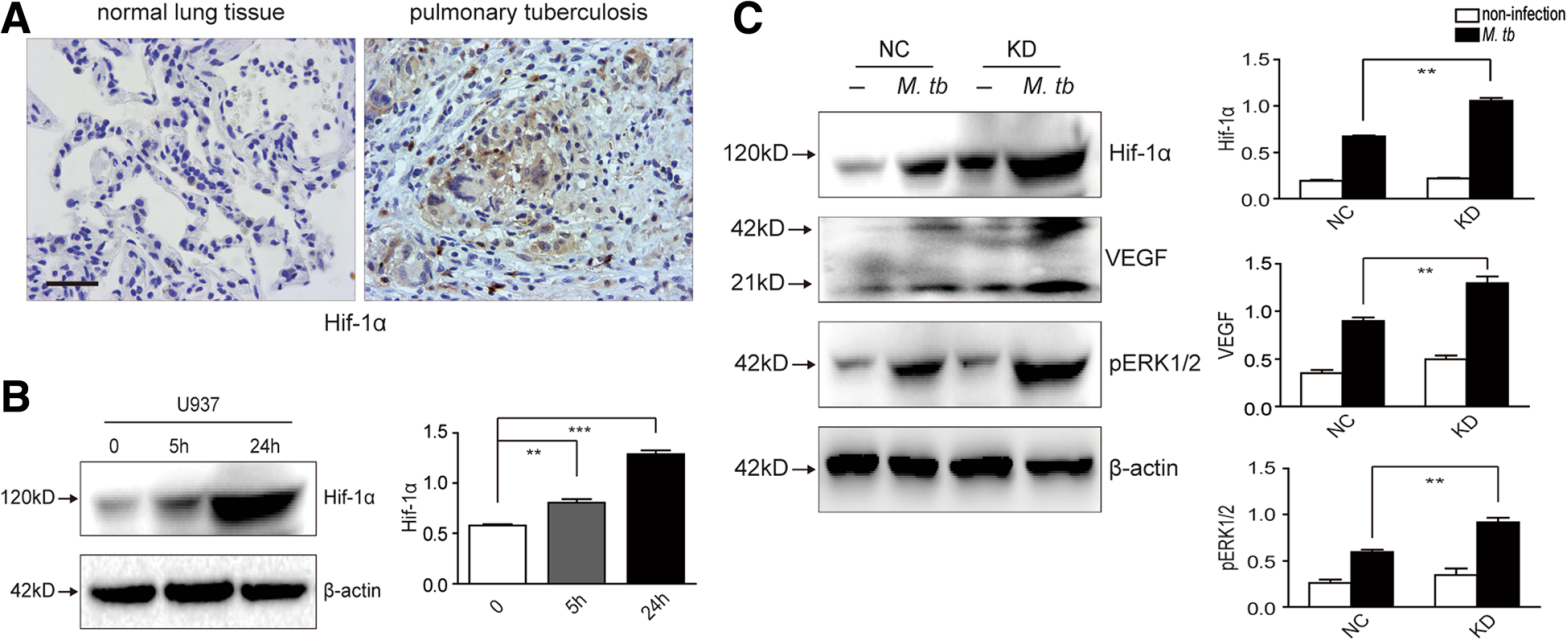
IRAK-M affected Hif-1 and MAPK signaling cascade during *M. tb* infection. **a** Human samples from pulmonary tuberculosis (*n* = 6) were collected for Hif-1α detection by immunohistochemistry (400 ×, scale bar 50 μm). Para-carcinoma tissues of human lung cancer (*n* = 6), were used as negative control. Representative images were shown (400 ×, scale bar 10 μm). **b** U937 cells were infected with *M. tb* (MOI 10) at indicated time and expression of Hif-1α was evaluated by Western blot. **c** Lentivirus infected U937 cells (NC and KD) were infected with *M. tb* (MOI 10) for 24 h and Western blot was performed to detect Hif-1α, VEGF and phosphorylated ERK1/2. Densitometric analysis was performed using pooled data from three such experiments. Data were mean ± SD (**, *p* < 0.01; ***, *p* < 0.001)

Hif-1 and MAPK signaling cascade were reported to be involved in bactericidal activities of macrophages [28–31]. We further investigated the expression of Hif-1α and MAPK in IRAK-M knockdown macrophages. It was shown that Hif-1α expression was increased in U937 cells as infected by H37Rv at 5 and 24 h post-infection (Fig. 5b). Histologically, in lung tissues of patients with pulmonary tuberculosis, it was also found that the expression of Hif-1α was increased (Fig. 5a). Interestingly, as IRAK-M was knocked down in U937 cells, the increase of Hif-1α induced by *M. tb* infection was much more obvious than in negative control cells (NC), in which IRAK-M was not knocked down (Fig. 5c). VEGF, a recognized downstream molecule regulated by Hif-1 [32], and the phosphorylation of ERK1/2 (pERK1/2) were also detected in IRAK-M knockdown U937 cells. It was shown that both 21 kDa monomer and 42 kDa dimer of VEGF as well as pERK1/2 were enhanced as IRAK-M was knocked down (Fig. 5c). Taken together, these results indicated that IRAK-M knockdown enhanced Hif-1 and MAPK signaling cascade during *M. tb* infection.

## Discussion

In the current work, the biological significance of IRAK-M, which is restrictively expressed in certain cell types such as monocytes/macrophages and plays a negative role in PAMP-TLR signaling pathway, was investigated in *M. tb* infection. Firstly, increased expression of IRAK-M was determined in *M. tb* infected macrophage cells and also in lung tissue of patients with pulmonary tuberculosis. As a negative regulator involved in innate immunity, along with induction of kinase activity of IRAK1/4 during infection, IRAK-M interacts with IRAK4 to induce transcription of downstream inhibitors such as A20, IĸBα, SOCS-1 and SHIP, to prevent radical immune pathology of the host [14, 18]. As shown in Fig. 4, CpG7909, the ligand of TLR9, induced IRAK4 increase. Meanwhile, IRAK-M was also increased, which is consistent with the description that IRAK-M plays a balanced role in IRAK1/4 activity. Interestingly, during *M. tb* infection, IRAK-M continued to increase, while IRAK4 increase induced by CpG7909 was suppressed. The biological significance of the IRAK-M increased to the intracellular survival of *M. tb* was further investigated in IRAK-M knockdown and over-expression cell systems. We found that the IRAK-M expression was beneficial for *M. tb* intracellular survival and proliferation. IRAK-M might be utilized by intracellular bacteria such as *M. tb* to act as their in vivo shelter.

We suspected that intracellular survival of *M. tb* affected by IRAK-M expression level might be related with macrophage polarization to M1 or M2 phenotypes. Functionally, M1-type macrophages were highly bactericidal, while M2 were active in anti-inflammation, promoting phagocytosis and tissue-repair [33]. Investigations in U937 cells indicated that *M. tb* infection induced both M1- and M2-type polarized macrophages. However, in *M. tb* infected IRAK-M knockdown U937 cells, M1-type polarization of U937 was enhanced, while M2-type polarization of U937 was inhibited. We further used an immunostimulant, CpG7909, to direct U937 cells firstly into M1 status and then infected cells with *M. tb*. CpG (Cytosine-phosphate-Guanine) is a ligand of TLR9, which is able to induce innate immune responses and also strong Th1-type immunity [34, 35]. CpG7909 is a relatively mature and safe CpG, which has been used in clinical treatment of cancer, allergies, asthma and also used as vaccine adjuvant of hepatitis B, malaria [36, 37]. In our previous work, CpG7909 was used as adjuvant of an anti-tuberculosis subunit vaccine. CpG7909 was able to enhance Th1-type immunity induced by subunit vaccine [38]. In the current work, it was determined that, as immunostimulant, CpG7909 induced M1 polarization of U937 cells, however, *M. tb* infection suppressed CpG7909-evoked M1 polarization. Strikingly, as IRAK-M was knocked down, the suppression of CpG7909-induced M1 polarization due to *M. tb* infection, was reversed, implying that IRAK-M facilitates *M. tb* intracellular survival via inhibiting M1-type or promoting M2-type polarization of macrophages.

We have previously reported that CpG7909 was able to enhance the ability of subunit vaccine to induced Th1-type immunity, however, CpG7909 was not able to improve its protective effects against *M. tb* infection [38]. The negative regulatory role of IRAK-M might contribute to this phenomenon. IRAK-M plays a negative regulatory role in most of TLR signaling pathway, including TLR1, TLR2, TLR4, TLR5, TLR6, TLR7, TLR8 and TLR9 [15]. New type adjuvants such as CpG DNA, which is developed from TLR ligands, are able to induce Th1 immunological responses of the host via IRAK1/4 signaling, which might be simultaneously balanced by IRAK-M. During *M. tb* infection, *M. tb* might utilize IRAK-M, a negative regulator located upstream in TLR signaling, to reverse the host’s immunity into their favorable status, which suppresses the effect of anti-tuberculosis vaccine.

MAPK (mitogen-activated protein kinase) and Hif-1 (Hypoxia inducible factor 1), an important transcriptional factor that mediates cellular responses to hypoxia and stressors in chronic infection, were reported to regulate bactericidal activities of macrophages, through iNOS or NFκB expression [28–31]. During *M. tb* infection, relative hypoxia of human tuberculous granulomas contributes to *M. tb* latent infection phenotypes and the associated resistance of *M. tb* to host and pharmacological killing [39, 40]. Recently, it was reported that Hif-1 regulates the shift of energy metabolism of macrophages activated by IFN-γ into aerobic glycolysis and the positive feedback loop between Hif-1 and glycolysis reinforces activation of macrophages and control of *M. tb* infection [41].We have previously reported that activation and phosphorylation of MAPK ERK1/2 prevents Hif-1α from ubiquitination and promotes the consequent nuclear import of Hif-1 complex [42]. In the current work, it was shown that during *M. tb* infection, both in infected U937 cells and in human tissue of pulmonary tuberculosis, Hif-1α obviously increased. Surprisingly, as IRAK-M was knocked down, expression of both Hif-1α and MAPK was increased, indicating that IRAK-M was involved in regulating Hif-1α and MAPK signaling cascade during *M. tb* infection. Both MAPK and Hif-1 are nuclear factors, transcriptionally inducing expression of downstream molecules, to regulate cell’s adaptive responses to infections or other stress factors [30, 43]. The mechanism of IRAK-M affecting MAPK and Hif-1 signaling cascade, and whether MAPK and Hif-1 are involved in IRAK-M directing macrophage’s polarization, remains further research. Furthermore, the mechanism, how IRAK-M acts as access board for intracellular bacteria such as *M. tb* to deviate polarization of macrophages, delay antigen-presentation and bactericidal activity of macrophages, resist Th1-type anti-tuberculosis immunity of the host, will be worthy of further investigation.

## Conclusion

In summary, the role of IRAK-M in polarization and function of macrophages, intracellular survival of *M. tb*, was investigated in this study, which might be helpful to understand the significance of IRAK-M to pathogenesis and host-pathogen relationship of *M. tb* and meaningful to resolve the dilemma of development of anti-tuberculosis vaccine.

## Abbreviations

AAM: Alternatively activated macrophages
AF: Acid-fast
Arg-1: Arginase-1
CAM: Classically activated macrophages
CFU: Colony forming units
CpG: Cytosine-phosphate-Guanine
DAPI: 4,6-diamidino-2-phenylindole
Eni.S: Enhanced infection solution
ERK: Extracellular regulated protein kinases
Hif-1α: Hypoxia-inducible factor-1α
IF: Immunofluorescence
IFN-γ: Interferon gamma
iNOS: inducible nitric oxide synthase
IRAK: IL-1 receptor-associated kinase
JAK: Janus kinase
KD: Knock-down
LV: Lentivirus
*M. tb*: *Mycobacterium tuberculosis*
MAPK: Mitogen-activated protein kinase
NC: Negative control lentivirus vectors
OE: Over-expression
PAMP: Pathogen-associated molecular pattern
PBS: Phosphate-buffered saline
PMA: Phorbol myristate acetate
PRRs: Pattern-recognition receptors
pSTAT: phosphorylated STAT
STAT: Signal transducers and activators of transcription
TLR: Toll like receptor
VEGF: Vascular endothelial growth factor
